# Exploring Research on the Drug Loading Capacity of Quantum Dots

**DOI:** 10.7759/cureus.67869

**Published:** 2024-08-26

**Authors:** Kevin Jordan Noel, Marakanam S Umashankar, Damodharan Narayanasamy

**Affiliations:** 1 Department of Pharmaceutics, SRM College of Pharmacy, Faculty of Medicine and Health Science, SRM Institute of Science and Technology, Chengalpattu, IND

**Keywords:** toxicity, regulatory framework, fret, nanoparticles, biomedical applications, drug delivery, quantum dots

## Abstract

Quantum dots (QDs), also known as quantum nanodots or colloidal nanocrystals, possess unique visual and electrical properties that have enabled various applications in biomedicine, particularly in drug delivery. Quantum dots offer significant advantages, such as a high surface area for drug attachment, the ability to modify solubility and drug release patterns, and the potential for targeted delivery. This review covers various aspects of QD research, including their synthesis, properties, and the challenges associated with their use. Key challenges include concerns about QD toxicity, stability, and environmental impact. Additionally, the article discusses using quantum dot-Förster resonance energy transfer (QD-FRET) to study in vivo drug release kinetics. This capability is essential for evaluating the performance of QDs as drug carriers and understanding their interactions within biological systems. In summary, while QDs present promising opportunities for advancing drug delivery mechanisms, ongoing research is necessary to mitigate toxicity concerns and enhance their biocompatibility, paving the way for their clinical application in targeted therapies.

## Introduction and background

In recent years, there has been substantial growth in scientific studies, especially in the fields of nanoscience and nanotechnology. Nanotechnology produces nanoparticles, which include a variety of forms such as fullerenes, dendrimers, carbon nanotubes, metallic nanoparticles, liposomes, and quantum dots (QDs). It offers significant flexibility and advantages in biomedicine for both diagnosis and therapy. Nanostructured materials serve as a link between the macroscopic world and the molecular scale, opening novel possibilities for applications in electronics, biology, and optoelectronics. The ability of nanocarriers to perform multiple functions, including drug residence ligand affinity, molecular imaging, and traceable delivery of drugs, is advantageous in the field of nanotechnology [1].

The pharmaceutical industry currently offers a wide variety of formulations, but due to reduced bioavailability due to low water solubility and cell membrane permeability, their effectiveness is limited. Conventional formulations feature shortcomings such as toxic behaviour, poor dissolution, and inappropriate release of drug patterns [2].

Nanotechnology, an innovative drug delivery approach utilising intelligent nanocarriers with precise shapes and sizes, can address these challenges and facilitate the development of safe and efficient nanomedicines [3].

Nanotechnology-based formulations offer a straightforward means to modify the drug's solubility and its targeted release at the disease site and minimise non-specific toxicity. Nanotechnology, employing molecules at the nanoscale, possesses distinctive characteristics like self-assembly, stability, biocompatibility, specificity, and drug encapsulation, attributed to its material composition [4].

Among the various nanocarriers used for chemotherapy, quantum nanodots, which are colloidal nanocrystals, are chosen owing to their unique characteristics. Ekimov and Onushenko described QDs, a type of nanoscale semiconductor crystal, in matrix glass and reported their imaging use in 1998 [5].

Quantum dots are semiconductor nanoparticles made by assembling groups IIeVI or IIIeV that are more diminutive typical exciton Bohr radius in the bulk material (2e10 nm). These nanocrystals exhibit intriguing optical and electronic traits, resulting in extensive development across various applications such as disease diagnosis, single protein tracking, drug delivery, intracellular therapy, and more. Quantum dots are widely utilised in clinical settings due to their size and unique properties. Their exceptional physical, optical, and electrical characteristics make them valuable in various applications beyond clinical use, including analysis, imaging techniques, biomaterial regeneration, oncology treatment, photothermal therapy, biosensing, biodefense, and medication administration [6].

Utilising large-scale particles for drug administration has drawbacks, including instability in vivo, low solubility, reduced availability, inadequate uptake, and potential side effects. In conclusion, QDs are better at transporting drugs because they can easily pass through cell membranes and have a larger specific surface area, which makes drug targeting more flexible. The primary function of these applications lies in their capacity as intelligent nanocarriers for drug delivery, enhancing the effectiveness of pharmacological treatments. They enable monitoring of the drug's impact on the body and facilitate the creation of novel medications for addressing emerging diseases. The core-shell structure has expanded research opportunities in various domains, including optics, catalysis, electronics, biomedical, pharmaceutical, and drug delivery. Encapsulating QDs in a shell made of noble metals or conductive polymers can improve their conductivity and electrical characteristics [7].

This leads to higher performance through a synergetic effect between the core and shell. Quantum dots have a rigid structure that offers a significant surface area for drug attachment. In this process, the drug is not enclosed within the QDs but rather adheres to the surface through adsorption or binding to existing bonds on the QD. Quantum dots have two functional groups that can bind drugs: free carboxylic acid (COOH) and free amine (NH2). The choice of QDs is determined by their intended applications across various fields. Quantum dots typically have a semiconductor outer shell composed of mineral compounds such as cadmium, selenium, zinc oxide, silicon dioxide, etc. [8].

The surface coating provides a particular site for conjugation and lowers toxicity. Biocompatible QDs, such as carbon, graphene, and zinc oxide, are employed in drug delivery systems to enhance aqueous solubility. Carbon QDs are recommended for delivering mitomycin, an anticancer drug. Medications can be incorporated into QD nanocarriers through dissolution, dispersion, adsorption, and coupling methods. This process alters the physical and chemical attributes of the drugs (e.g., equilibrium solubility, dissolution speed, lattice structure, surface water-repellence, and water-attracting property), as well as their physical responses and biological properties, influenced by the carriers and consequently, drug assimilation, dispersion, chemical processing, and expulsion [9].

**Figure 1:**
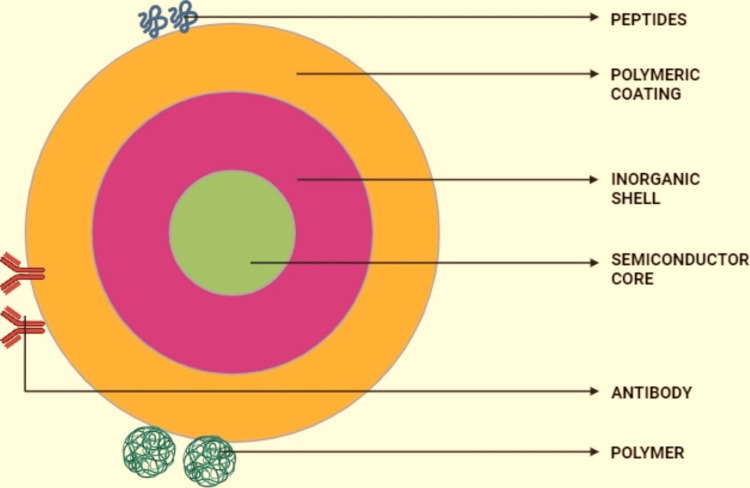
Structure of quantum dots using bioactive agents This image is created by the original author,  Kevin Jordan Noel.

Drug nanocarriers primarily consist of nanotransporters, lipid-based carriers, and other types, polymer nanostructures or nanocontainers, microemulsions or nanosized emulsions, nanomicelles, dendrimeric structures, inorganic nanotransporters like silica nanobeads, nanotubes, and QDs. Ultimately, QD nanocarriers for pharmaceuticals have the potential to boost effectiveness, minimise side effects, and enhance the therapeutic index of medications. Furthermore, drug nanocarriers can enhance the absorption of small-molecule drugs efficiently. Simultaneously, studies on macromolecular drug delivery have shown promising potential as well. QD labelling advances research into nano-drugs at the cellular level, including in living animals. Surface modifications with targeted ligands are widely employed to improve medication delivery efficiency. After nearly a decade of progress, advancements have been achieved in the technology of surface modifications of QDs. Surface-bound ligands like mercaptoacetic acid, mercaptoethylamine, polyethene glycol, and hydrophilic polymer chains containing carboxylic groups are now utilised. These ligands can attach drug bioactive compounds to QDs via charge-based interactions or chemical bonds, forming nanocarrier drug complexes. This enables the phosphorescent tracking of therapeutic agents within biological units or fauna, showcasing the potential for precise drug delivery and monitoring in biomedical applications. Figure 1 showcases the QD structure. This generation's latest water-soluble QDs have addressed various issues such as decreased production, susceptibility to chemical reactions, and limited shelf stability encountered in colloidal nanocrystals produced through ligand-swapping solubilisation techniques. Cadmium-selenium and cadmium-tellurium QDs exhibit enhanced biological uses. These QDs demonstrate a clear connection between their dimension, stimulus energy, and radiated wavelength [1].

## Review

Properties and characteristics of quantum dots

**Figure 2:**
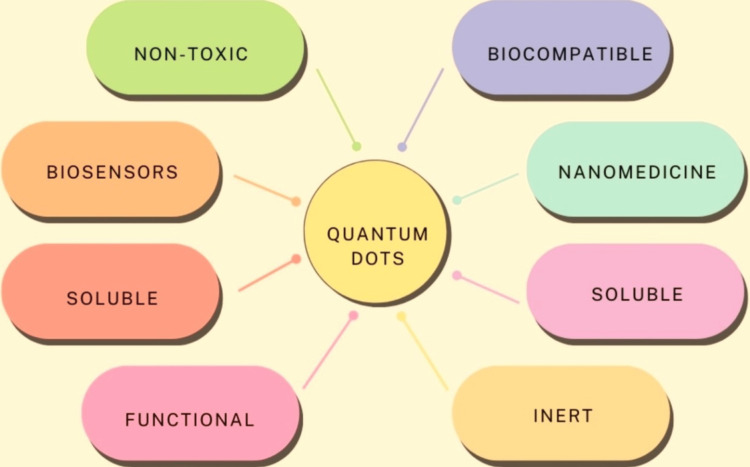
Properties of quantum dots This image is created by the original author, Kevin Jordan Noel.

Quantum dots possess many aspects, including size-modifiable light emission, enhanced signal luminosity, resilience against light-induced fading, and concurrent multi-phosphorescence emissions, due to their highly reactive surface, they must be used under ambient circumstances after being stabilised and passivated, due to their ability to emit multiple fluorescent signals, this material can be integrated into a traceable drug delivery system, enhancing the pharmacokinetic and pharmacodynamic effects of medications and when creating a biological substance which functions as an enhancement to pharmaceutical administration applications, quantum nanodots can serve as a template for a carrier that fits in dimensions and aspects. The properties of QDs are shown in Figure 2.

Virtues of quantum dots

There is a maximum lifespan for QDs, and they are less susceptible to deterioration. The tiny size of QDs makes them versatile, enabling their incorporation into diverse settings like fabrics and polymer scaffolds. Augmented optical behaviour is utilised in bioengineering and biosciences. Cadmium selenide (CdSe) QDs are substantially brighter, approximately twentyfold more luminous than fluorescent markers.

Drawbacks of quantum dots

Throughout the production process, it can be challenging to regulate the size of colloidal nanocrystals accurately. When a polymer shell exists, QDs diminish some of their optical characteristics [4].

Types of quantum dots

Classification Based on Composition

Conventional QDs: Conventional QDs usually have a core-shell design with a core made of semiconductor material and a shell surrounding it, which improves stability and optical characteristics. Conventional QDs are commonly used in various fields, like light-emitting diodes (LEDs) and biological imaging, and provide adjustable and luminous emission characteristics.

Core-coating QDs: Core-shell nanocrystals are engineered with a core material encapsulated by a shell layer to control emission wavelength and enhance quantum efficiency. These QDs are preferred for applications in quantum computing and sensing technologies due to their controlled emission properties.

Alloyed QDs: Alloyed QDs, composed of mixed semiconductor materials, offer tunable emission spectra and high colour purity by blending different components. Their applications in display technologies and photovoltaics underscore their efficiency in light absorption and energy conversion.

Additional Compositional Classes

Quantum dots can also be categorised based on the specific elements they contain, which include the following:

Group II-VI QDs: They are composed of components from the second and sixth groups of the periodicity chart, such as CdSe, cadmium sulphide (CdS), and zinc sulphide (ZnS).

Group III-V QDs: They are made from components from the third and fifth groups of the periodicity chart; examples include indium phosphide (InP), indium arsenide (InAs), and gallium arsenide (GaAs).

Transition metal dichalcogenide QDs: These consist of transition metal atoms and chalcogenide atoms, such as molybdenum disulfide (MoS_2_), tungsten disulfide (WS_2_), and molybdenum diselenide (MoSe_2_).

Perovskite QDs: They are characterised by a structure similar to that of perovskite materials, typically formulated as ABX3, in which the cations are univalent (A), divalent metal ions (B), and halide ions (X).

This classification based on composition not only helps in understanding the fundamental properties of quantum dots but also guides their synthesis and application across different technological and scientific domains.

Dimensional classification of quantum dots

Heterostructure Categories

Quantum dots can be grouped into four main heterostructure categories: class I, inverted class I, class II, and inverted class II. Each class is defined by specific alignment and interaction of the conductivity and valency bands between the nucleus and encasing materials, impacting their electronic and optical properties. The construction of these heterostructures often involves the deposition of an outer shell material, which can introduce physical strain on the quantum dot lattice. This strain can limit the thickness of the shell that can be grown without diminishing the photoluminescent performance of the QDs.

Dimensional Constraints

The classification of QDs based on dimensionality distinguishes between 0-dimension (0D), one-dimension (1D), two-dimension (2D), and three-dimension (3D) structures, each confined at the quantum level.

0-dimension QDs: These are confined to all three spatial dimensions, typically manifesting as nanoparticles.

One-dimensional QDs: They are confined to one dimension; these structures include nanowires and nanorods.

Two-dimensional QDs: These are confined to two dimensions, known as quantum wells.

Three-dimensional QDs: These structures are not confined in any dimension and resemble bulk materials [5].

Challenges involving the use of quantum dots 

Quantum dots in biological research often face challenges related to their solubility in water and surface functionalization. Synthesis methods, while varied, often involve complex and potentially environmentally harmful processes. The synthesis of cadmium-based chalcogenide QDs is complex and expensive, which hinders their commercialization. Additionally, the use of harmful solvents in synthesis methods poses a major obstacle to minimising environmental impact. Concerns about stability are particularly important for perovskite QDs, as they face instability problems that restrict their real-world usage. Toxicity is a significant issue, particularly for QDs that have heavy metals such as lead or cadmium, which present risks to both health and the environment. Efforts to reduce harmful effects, like finding alternatives to lead and creating core and shell structures, are being investigated but need more advancement. Quantum dots have shown promise in the realms of drug delivery and bioimaging due to their unique optoelectronic properties. However, challenges like biodistribution, toxicity, and long-term stability in biological buffers impede their possible applications [1]. [6] investigated surface alterations and developments in QDs to tackle these obstacles. Quantum dots are known for their exceptional optical characteristics, which have resulted in their utilisation in diverse biomedical and pharmaceutical fields. Nevertheless, worries regarding their possible harmful effects on health and the environment continue to exist. These worries stem from the makeup of QDs, which frequently include toxic heavy metals such as cadmium or lead. Even though QDs have great promise, the existence of toxic heavy metals like cadmium presents a major hurdle for their use in medicine. Research has demonstrated that QDs can greatly impact the functioning of mitochondria, causing disruptions in bioenergetics and triggering mitochondrial permeability transition, a sign of cell death. Moreover, there are potential inhalation hazards associated with the aerosolization of QDs in manufacturing processes, which highlights the importance of implementing safety measures early on during research and prototyping activities. In a contradictory manner, progress in creating and changing QDs is focused on reducing these safety risks. Creating core-shell structures with biocompatible ligands and producing heavy metal-free and metal-free QDs are effective ways to minimise toxicity. Despite attempts made, the translation of QDs into products suitable for clinical use is obstructed by safety issues, requiring further research to address these obstacles. [7] highlighted the potential of QDs in nano-based drug delivery for pharmaceutical and biomedical applications, as well as the current challenges in their clinical integration, and reviewed the experimental and theoretical challenges in measuring and interpreting the energy spectra of few-particle QDs, particularly in terms of the interplay between single-electron confinement, cyclotron, and electron-electron interaction energies. The challenges in QD research are multifaceted, highlighting the need for precise control over QDs' electronic and optical properties, a challenge that is echoed by further emphasising the importance of surface modifications for specific applications while underscoring the need for a better understanding of the robustness and stability of QD photovoltaic devices. These challenges collectively underscore the complexity of QD research and the need for continued innovation in this field. The creation of QDs comes with numerous obstacles in their production, doping, and utilisation in different areas. One of the main difficulties when creating colloidal QDs, like cadmium selenide, is ensuring consistent size and composition, which is essential for their electronic characteristics. In the same manner, doping semiconductor QDs face challenges such as dopant clustering and their removal from the lattice, which can impede achieving consistent doping and desired qualities. Additionally, combining QDs with drugs for specific treatments brings up issues regarding the best size and stability, along with potential toxicity in both the long- and short-term, which must be identically resolved for safe use in living organisms. Careful consideration of the composition, morphology, and optical behaviour of QDs is necessary for achieving the desired photoactivity in photocatalytic applications. Furthermore, microfluidic reactors, despite providing precise control over particle characteristics, face challenges in terms of scaling up and ensuring consistent production of QDs. The metabolism and breakdown of QDs in the body are not well understood, and research has indicated that QDs can build up in the kidney, spleen, and liver. It is also uncertain if the body can eliminate the QDs. Quantum dots cause aggregation in live cells, potentially disrupting cell function. There is also a challenge in attempting to introduce QDs into cells without causing harm to the cells during the delivery process. Even though QDs are tiny, attaching them to various molecules through bioconjugation can enlarge their size and hinder their delivery into cells. Challenges in QD-based devices, such as light-emitting diodes and displays, involve heavy metal usage, high hole injection barriers, and improving printing processes for pixilation. Moreover, the production of III-V QDs through solution-phase synthesis is currently in progress. Still, their quality is not as advanced as that of II-VI and IV-VI QDs because of challenges in selecting precursors and regulating nucleation and growth processes. Finally, addressing the fabrication process, stability, and catalytic efficiency issues is crucial regarding the development of nanostructure-based polymer blends for catalysis. In summary, the formulation of QD nanocrystals is a complex process that necessitates addressing challenges related to synthesis, doping, stability, toxicity, and integration with other materials for specific applications. Overcoming various obstacles is necessary for the development of QD technologies for the efficient implementation of practical devices and therapies [1].

Quantum dots in the pharmaceutical field

Metallic nanoparticles have found applications in the fields of screening, radiography, and medication transport. In the modern era, biosensors have been developed that leverage the photonic properties of gold (Au) as well as the radiance of colloidal nanocrystals, known as QDs. These QDs offer valued contributions to pharmaceutical transport, radiography, and alternate applications in pharmaceutical research. The fluorescence seen in QDs can aid in the detection of active metabolites, revealing new avenues of research [1].

Delivery of drugs through quantum dots

Quantum dots, owing to their small size (typically 5-10 nm), exhibit a unique property that enables them to evade renal clearance and be effectively taken up by the reticuloendothelial system. This characteristic enhances their circulation time and efficiency, making them suitable for multifunctional applications, including imaging and drug delivery, which holds significant promise for targeted therapy and improved treatment outcomes. Quantum dots, coated with water-soluble capping agents such as mercaptoacetic acid and polyethylene glycol (PEG) conjugates, can be conjugated to drugs through covalent bonds or electrostatic interactions. This allows for the formation of stable and targeted complexes that can facilitate the delivery of therapeutic agents to specific sites within the body. Quantum dots have been extensively developed and explored for their potential applications in the field of nanomedicine. They have been engineered to serve as targeted drug delivery vehicles, selectively transporting doxorubicin to cancer cells and emitting fluorescence signals to visualise cancer cell populations. This conjugation approach has been successfully applied to the imaging and therapy of prostate cancer, leveraging the unique properties of QDs to enhance diagnostic accuracy and therapeutic efficacy. To develop a theranostic strategy targeting cancer cells, paclitaxel, a widely used chemotherapeutic agent, was conjugated with CdSe and ZnS QDs. The resulting nanoparticles exhibited an encapsulation rate of approximately 80% and a drug loading rate of 4.7%. In vitro studies demonstrated that these nanoparticles effectively inhibited tumour cell growth by 78%, highlighting their potential for targeted cancer therapy and imaging applications. Table 1 regarding the catalogue of QDs has been mentioned below. Graphene QDs have emerged as a prominent choice for drug delivery applications among various types of QDs. This is due to their unique properties, including chemical inertness, biocompatibility, and low toxicity, which render them an ideal carrier for delivering therapeutic agents. These attributes make graphene QDs a promising platform for the development of targeted and efficient drug delivery systems [1].

**Table 1:** Catalogue of quantum dots used in drug delivery PEG: polyethylene glycol

Drug	Quantum dot	Treatment	References
Doxorubicin hydrochloride (DOX)	Chitosan-encapsulated zinc oxide (ZnO)	Leukaemia	[8]
Adriamycin	Graphene oxide quantum dots conjugated with folic acid	Cervical cancer	[9]
Doxorubicin	Water soluble graphene quantum dots	A549 cell: lung cancer	[10]
Doxorubicin, hyaluronic acid	Quantum dots with pH-sensitive ZnO	Lung cancer	[11,12]
Busulfan	Manganese-doped zinc sulfide (Mn-ZnS) nanocrystals	chronic myelogenous leukemia	[13]
Doxorubicin	Graphene quantum dots; magnetic silica nanocarriers	Breast cancer 4T1 cells	[14]
Gemcitabine	Graphene quantum dot-tagged human serum albumin nanoparticles	Pancreatic carcinoma	[15]
Methotrexate	Cadmium telluride (CdTe) nanocrystals embedded in nanogels	Skin inflammation or autoimmune arthritis	[16,17]
Doxorubicin cis-platin	Graphene quantum dots	Glioma cells	[18]
Bevacizumab ranibizumab	PEGylated graphene QDs encapsulated in beta (β)-cyclodextrin	Ocular disorders	[19]

Quantum dots as traceable transport of drugs

The photophysical aspects of colloidal nanocrystals enable their use in live monitoring of dynamics in nanocarriers and medication dispersion. This capability is crucial for optimising the efficacy and safety of nanomedicine-based therapies, as it allows for the tracking of nanocarrier distribution, drug release kinetics, and potential off-target effects in real-time. Homogeneous nanocrystals, specifically CdSe coated with ZnS nanoparticles, exhibit a unique property where they radiate strong light within a limited spectral wavelength, with the spectrum depending on the diameter of the nucleus. This size-dependent emission behaviour allows for precise control over the optical properties of these nanoparticles, making them valuable for various biomedical applications. The multifunctional nature of quantum dots, encompassing both imaging and illuminating capabilities, enables the simultaneous tracking of nanocarriers in biological systems and the observation of their behaviour under identical conditions. This multicolour approach allows for the real-time monitoring of nanocarrier dynamics and interactions within the biological environment, providing valuable insights into their efficacy and safety [1].

Utilising quantum dot nanocarriers for drug delivery

The integration of QD colloidal nanocrystals with nanocarriers has paved the way for investigation as well as the development of tailored physicochemical properties that optimise drug delivery applications without compromising the biological system or the drug's efficacy. This approach enables the design and optimisation of nanocarriers that can effectively deliver therapeutic agents while minimising potential adverse effects on the biological environment. Quantum dot nanocarriers can be loaded with drugs through various methods, including dissolution, dispersion, adsorption, and coupling. These techniques enable the efficient incorporation of therapeutic agents into the nanocarrier, allowing the creation of targeted and controlled pharmaceutical transport systems. The use of nanocarriers, such as QD nanocarriers, can alter the pharmacokinetic and pharmacodynamic properties of drugs, influencing their absorption, distribution, metabolism, and excretion (ADME). Despite this, QD nanocarriers have been shown to increase a medication's effectiveness and decrease its adverse effects, which will ultimately improve its safety margin [1].

Properties of quantum dots as nanocarriers

**Figure 3:**
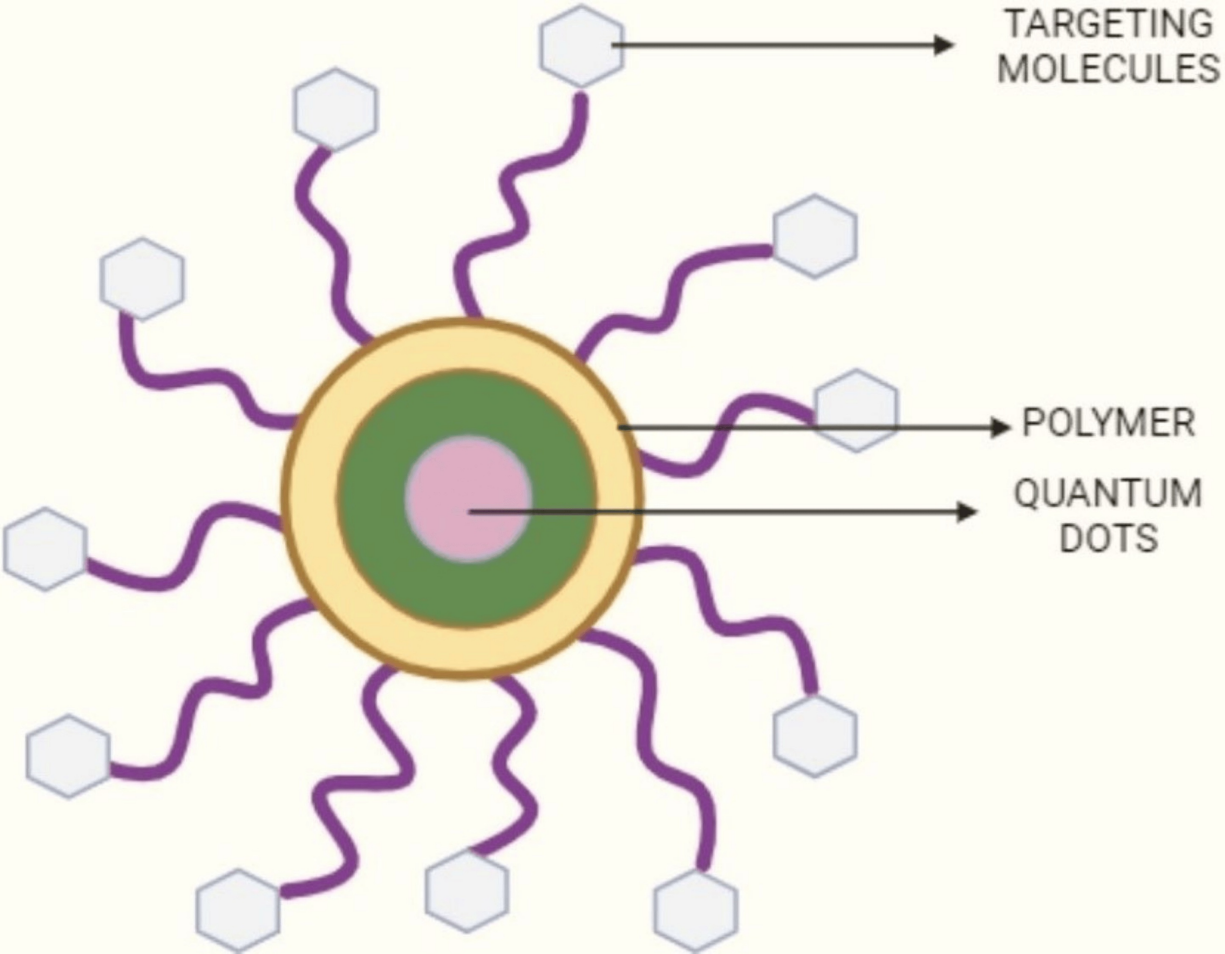
Quantum dots as a polymeric nanocarrier This image is created by the original author, Kevin Jordan Noel.

Nanocarrier materials containing QDs should contain the following characteristics: no drug interactions, more drug-loading capacity, low biocompatibility and toxicity, mechanical strength, long residence time in vivo, and improved stability and circulation time. When used as nanocarrier particles on the nanometre scale, QDs exhibit various effects, including quantum confinement, size, and dielectric effects. The diagram of QDs as polymeric nanocarriers is illustrated in Figure 3. These unique properties have significant implications for their biological and life sciences applications. Currently, various types of QD nanocarriers are being explored for cancer therapy, including lipid nanoparticles, nano-sized hydrogels, chitin-derived polymers, folate, and carbon nanostructures, each offering distinct advantages and potential for targeted therapeutic administration and diagnostics [1].

Quantum dot-based targeted therapy

Achieving targeted medication administration is possible through various strategies, including potential hydrogen (pH)-controlled, temperature-controlled, optical wave-mediated, and magnetic field-mediated releases. These approaches enable the precise and controlled delivery of therapeutic agents to specific sites within the body, enhancing efficacy and reducing side effects. Proteins and peptides can facilitate the penetration of cell membranes, allowing for the delivery of bi-conjugated QDs into cells. This approach leverages the natural ability of proteins and peptides to interact with cell membranes, enabling the targeted delivery of QDs for various biomedical applications. Intracellular delivery enhancers, primarily comprising biomolecular compounds, possess valuable tools for clinical use. Cationic QDs, in particular, enter cellular environments efficiently, while less significant QDs can traverse the nucleus, allowing for targeted delivery and potential therapeutic effects. Quantum dots conjugated with folic acid have been extensively studied and have been shown to bind specifically to folate receptors, which are overexpressed in many types of cancer cells. This targeted binding enables the QDs to accumulate in tumour cells, making them a promising tool for cancer imaging and therapy. Polymeric encapsulation, particularly chitosan-encapsulated QDs, can maintain the controlled release of the medication till the intended neoplastic cells become apparent. This encapsulation strategy ensures the drug is protected from premature release and degradation, allowing it to get to the desired site of action with optimal efficacy [1].

Advantages of using quantum dots in site-specific drug delivery system

Nanoparticle drug carriers have been engineered to target specific organs by altering biochemical systems. Drugs released from nanomaterials have a two-phase release profile, which is defined as an abrupt release at first and a continuous release after that over an extended period. This controlled release mechanism enables the delivery of a low dose of the drug in a manner that minimises side effects, thereby enhancing the therapeutic efficacy while reducing the risk of adverse reactions. Precision delivery of drugs enables the medication to remain localised at the targeted location over an extended period, thereby enhancing its absorption and bioavailability. This prolonged residence at the site of action facilitates optimal interaction between the drug and its target, leading to improved therapeutic efficacy and reduced systemic exposure, which in turn minimises the risk of adverse effects. The carrier molecule can modulate the membrane transport mechanism, thereby enhancing the drug's bioavailability by improving its water solubility and sustaining its release. This modification enables the drug to interact more effectively with its target site while also reducing the risk of rapid clearance and improving the overall therapeutic efficacy [1].

Quantum dots for drug release

**Figure 4:**
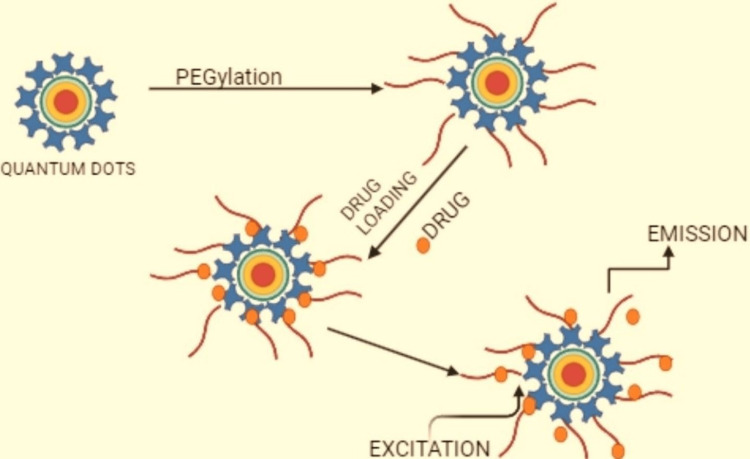
Synthesis of quantum dots of a PEGylated drug and its release This image is created by original author, Kevin Jordan Noel. PEG: polyethylene glycol

Liquid chromatography-mass spectrometry is a frequently used method for investigating the in vivo release of nanodrugs. Nevertheless, this approach is limited by the difficulty in distinguishing between free and encapsulated drug concentrations. To address this challenge, researchers have turned to quantum dots for Förster resonance energy transfer (QD-FRET), a promising technique for resolving this issue. By leveraging the singular characteristic of colloidal nanocrystals, researchers can accurately measure the release kinetics of nanodrugs, thereby providing valuable insights into their pharmacokinetic behaviour. The synthesis of quantum nanodots using PEGylated drugs and their release characteristics are shown in Figure 4. The fundamental principle underlying the QD-FRET method is based on the coinciding area of the fluorescence output range, the quantum dot, and the activation range of the fluorescence receptor. This coinciding area is critical, as it enables efficient energy transfer between the donor and acceptor molecules when they are nearby. The proximity of the acceptor and donor is crucial, as it facilitates the non-radiative energy transfer process, which is distance-dependent. The closer the distance between the acceptor and donor, the more efficient the energy transfer, thereby enhancing the selectivity and responsiveness of the QD-FRET method. In the QD-FRET system, the quantum dot serves as the donor, with its fluorescence emission spectrum acting as the energy source. This energy is then transferred to the acceptor molecule, which is typically a fluorescent receptor. The energy transmission from the light donor to the light acceptor leads to the quenching of the quantum dot's radiance and stimulation of the acceptor [1].

Quantum nanodots in therapeutic delivery

Lately, short inhibitory ribonucleic acid (RNA) therapy has garnered significant attention as a treatment for various human diseases, particularly carcinoma. However, the rapid degradation and clearance of short inhibitory molecules from the body pose substantial challenges. To tackle these challenges, scientists have investigated the utilisation of nanodelivery systems, including QDs, which have demonstrated potential in overcoming issues such as immune response, encapsulation efficiency, toxicity to cells, and cellular obstacles. Quantum dots, being capable of protecting biological drugs like short inhibitory RNA from degradation, offer a higher potency at moderate conditions. Furthermore, the development of quantum complexes, large nanotransporters formed by the accumulation of positively charged molecules and negative ions from quantum dot particles and deoxyribonucleic acid (DNA) polymers, enables the detection of small interfering RNA (siRNA) delivery in the cytoplasm, nucleus, and other cellular compartments [1].

Quantum dots for carcinogenic therapy

**Figure 5:**
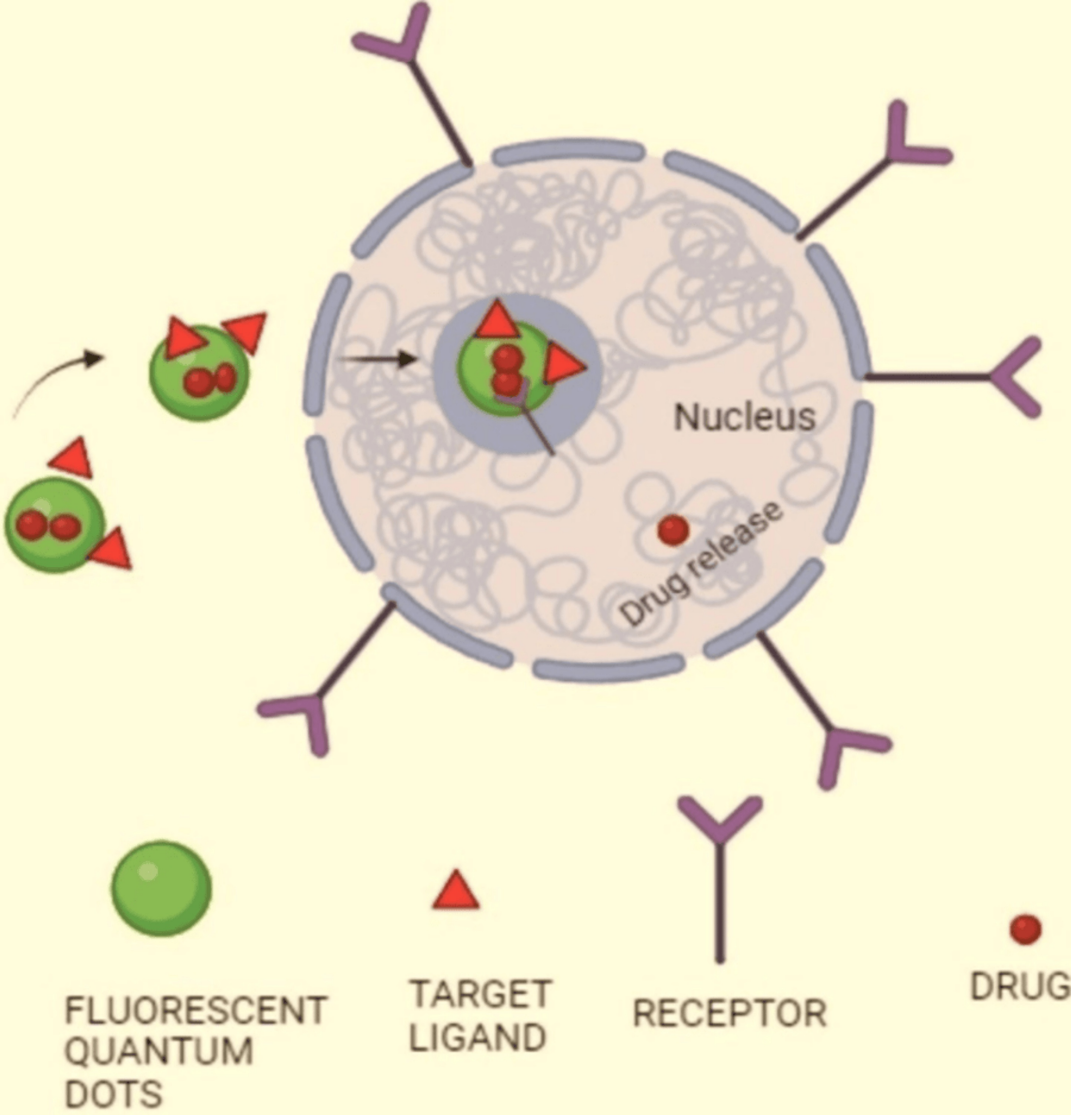
Drug delivery through quantum dots by receptor-mediated in cancer cells This image is created by the original author, Kevin Jordan Noel.

Quantum dot technology has significantly influenced cancer therapy, offering a promising approach to diagnosis and treatment. The unique properties of QDs, such as their tunable size, stable photoluminescence, and large surface-to-volume ratio, enable them to be a desirable platform for a range of uses. in cancer management. Sentinel lymph node (SLN) mapping is a critical stage in cancer surgery, as it identifies the main lymph nodes responsible for receiving metastases from the primary tumour. Predictive staging and the direction of surgical procedures depend on this technique. Through the process of mapping the SLN, surgeons are able to precisely remove the initial set of nodes that are most likely to contain metastases, guaranteeing complete removal of the tumour and reducing the likelihood of recurrence. The SLN is typically identified using a blend of approaches, including radiographic lymph mapping, indigo colourant, and an intraoperative radiation detector. Empirical evidence suggests that this methodology can augment the precision of lymph node staging, mitigate the necessity for supplementary lymph node dissections, and ameliorate overall patient outcomes. Earlier sentinel lymph node mapping techniques employed the use of radiotracers, such as technetium-99m (tc-99m), albumin colloids, and lymphazurin, for localization. However, these methods were limited by the potential for cellular damage, radiation dose, and the failure to visualise lymphatic tracers effectively. The precise identification and removal of SLN, which is essential for cancer surgery, was hampered by these restrictions. Quantum dots have been developed to improve the detection and resection of SLNs in cancer surgery. These nanoparticles offer enhanced light penetration through thick tissue, allowing for more accurate and minimally invasive SLN localisation. This improved penetration enables surgeons to minimise the size of surgical incisions, reducing patient morbidity and improving overall outcomes. Quantum dot application in SLN mapping has the potential to facilitate the complete removal of the primary tumour in a single surgical procedure. By providing enhanced visualisation and precise localisation of the sentinel lymph nodes, QDs enable surgeons to accurately identify and resect the lymph nodes most likely to harbour metastatic disease. Figure 5 shows how drugs are delivered to cancer cells via QDs mediated by receptors. Tumour-associated marker expression levels can be measured before and after cancer surgery using bioconjugated QD nanocrystals. This approach allows for the assessment of residual neoplastic cells that may remain following the primary surgical intervention. By utilising the unique optical properties and targeting capabilities of QDs, surgeons and clinicians can gain valuable insights into the tumour microenvironment and the presence of any residual disease [1].

Patent-related Information about quantum dots

Various patent databases, including Google's Intellectual Property Database, European Patent Office Search, Global Patent Registry, and US Patent and Trademark Office (USPTO), were searched to gather information on QDs. The search terms used included "quantum dots," "formulation of quantum dots," and "biomedical application of quantum dots." The search results were filtered to focus on relevant information, such as the title, abstract, and status of the studies, with a preference for patents written in English. Patent information on QDs has been mentioned in Table 2. Many patents have been granted for QD technologies in recent years. From 1999-2004, the USPTO issued 146 patents on QDs, with the top assignees being the Massachusetts Institute of Technology (MIT), the University of California, and Quantum Dot Corporation (QDC). The QDC has licensed 22 patents and owns or has licensed over 90 United States and international patent applications currently under examination, claiming exclusive licenses to key patents on QDs for biological applications. Some notable QD patents include a method for manufacturing QDs with a gradual composition gradient shell structure, using a nanofluidic channel to isolate, detect, and identify individual QD conjugates, and atomistic QDs with mechanically coupled dangling bonds that can be controllably perturbed to induce tunnelling and alter the electrostatic potential. The rapid growth in QD patents has led to a potential for overlapping and conflicting patents in the field, as different examiners may have reviewed different prior art. This lack of standardised terminology and the potential for patent conflicts may present challenges as QD technology continues to advance.

**Table 2:** Patent information on quantum dots

Patent number	Title	Publication date	Reference number
US20120009223	Collagen-based hydrogels incorporating ZnO Quantum Dots/pDNA complexes as corneal substitutes (plasmid deoxyribonucleic acid)	January 12, 2012	[20]
US20110287557	Single quantum-dot-based aptameric nanosensors	November 24, 2011	[20]
US20110278554	Hybrid quantum dot/protein nanostructures, methods of creation and application	November 17, 2011	[20]
US20110270153	Utilizing quantum dots for cell stimulation	November 3, 2011	[20]
US20110269297	Method for synthesising semiconductor quantum dots	November 3, 2011	[20]
US20110260111	Quantum dots, production methods, and usage methods	October 27, 2011	[20]
US20110260109	Water-soluble nanocrystalline quantum dots	October 27, 2011	[20]
US20110241229	Encapsulated nanoparticles	October 6, 2011	[20]
US20110237862	Multifunctional Fe3O4 (ferrous-ferric oxide) 0-cored magnetic-quantum dot fluorescent nanocomposites for cancer cell rf nanohyperthermia	September 29, 2011	[20]
US20110217721	Water soluble fluorescent quantum carbon dots	September 8, 2011	[20]
US20110129420	Materials and methods for biological imaging	June 2, 2011	[20]
US20110105643	Polymer-encapsulated nanoparticles	May 5, 2011	[20]
US20110086338	Bacteriophage/quantum-dot (PHAGE-QD) nano complex to detect biological targets in clinical and environmental isolates	April 14, 2011	[20]
US20110045094	Encapsulated quantum dot	February 24, 2011	[20]
US20110033954	Biofunctionalized quantum dots for biological imaging	February 10, 2011	[20]
US20100316797	Forming glutathione-capped and metal-doped zinc selenide/ zinc sulfide core-shell quantum dots in aqueous solution	December 16, 2010	[20]
US20100283037	Core-shell quantum dot fluorescent fine particles	November 11, 2010	[20]
US20100105089	Quantum dot biotags	April 29, 2010	[20]
US20100086993	Cell detecting system and quantum dot measuring system	April 29, 2010	[20]
US20100075361	Methods of generating fluorescence energy transfer (FRET) between semiconductor quantum dots and fluorescent dyes/proteins via multi-photon excitation, achieving zero background or direct excitation contribution to the FRET signature	March 25, 2010	[20]
US20090286257	Water soluble nanocrystalline quantum dot capable of near-infrared emissions	November 19, 2009	[20]
US20090200486	Quantum dot deoxyribonucleic acid metallic nanoparticle ensemble as fluorescent nanosensor system for multiplexed detection of heavy metals	August 13, 2003	[20]

Regulatory view and aspects of quantum dots

The regulatory framework for novel technology products is contingent upon their specific application and label claims, as governed by the relevant authorities. Notably, the regulatory landscape for these emerging technologies remains in a state of ongoing development.

FDA approval of quantum dots 

The FDA's viewpoint on QDs emphasises the importance of ensuring the safe and effective integration of nanotechnology into medical applications, particularly in drug delivery and diagnostic fields. The agency's regulatory science program is dedicated to developing methodologies and tools to evaluate the safety, characterisation, and effects of nanomaterials, including QDs. This initiative aims to create a comprehensive framework for assessing potential risks linked to QDs, such as toxicity and environmental impact while encouraging innovation in nanotechnology-based products. The FDA has released various publications detailing the applications of QDs in drug delivery systems, highlighting their capacity to enhance targeting and effectiveness in cancer treatments. Additionally, the agency collaborates with academic institutions and other governmental bodies to deepen the scientific understanding of QDs and their interactions within biological systems. As QDs progress in the pharmaceutical sector, the FDA's regulatory oversight will be vital in addressing safety issues and promoting the responsible advancement of nanotechnology-based therapeutics and diagnostics, ultimately safeguarding public health while fostering innovation in this promising area. The United States Food and Drug Administration has authorised a clinical study of the use of QD technology in human subjects. This marks the first instance of the FDA approving the use of inorganic materials for therapeutic purposes akin to a pharmaceutical drug. The trial, carried out by scientists at the University of Cornell, entails the therapy of five individuals with melanoma at Memorial Sloan Kettering Cancer Centre (MSKCC) to assess the safety guidelines and efficacy of QD nanocrystals [1].

Future perspectives

The future perspective of QDs in biomedicine looks promising yet complex. With their distinct visual and conductivity traits, QDs are poised to revolutionise drug delivery systems by offering high surface areas for drug attachment, customisable solubility, and targeted delivery capabilities. They enable advanced techniques like Förster resonance energy transfer for precise drug release monitoring. However, challenges such as toxicity, stability, and environmental impact remain significant hurdles. Advances in biocompatibility, regulatory approvals, and innovative synthesis methods will be crucial for integrating QDs into mainstream medical applications. As the regulatory landscape evolves, with milestones like the FDA's approval of QDs for melanoma treatment, the potential for QDs to enhance therapeutic efficacy and enable novel treatments for emerging diseases is immense. Addressing these challenges will be key to realising the complete ability of QDs in the future of medicine.

## Conclusions

Pharmaceutical delivery and imaging are two key areas where QDs have shown significant promise in the biomedical field. Their large surface area for drug attachment, adjustable optical characteristics, and customisable solubility make them an attractive platform for various therapeutic applications. As advancements in nanotechnology continue to evolve, the future of QDs appears promising. Researchers are exploring innovative applications beyond those currently in development, including their use in quantum computing, where they can serve as qubits, and in environmental monitoring, where they can detect pollutants at deficient concentrations. The potential integration of QDs into wearable technology and smart devices also opens new avenues for personalised healthcare and real-time monitoring of health conditions. In conclusion, the future of QDs is characterised by their versatility and potential to impact various sectors significantly. As challenges are addressed and new applications are discovered, QDs are likely to play a pivotal role in shaping the future of technology, healthcare, and environmental sustainability, making them a focal point for ongoing research and development efforts.

## References

[1] Jain NS, Somanna P, Patil AB (2021). Application of quantum dots in drug delivery. Nanosci Nanotechnol - Asia.

[2] Ekimov AI, Onushchenko AA (2023). Quantum size effect in three-dimensional microscopic semiconductor crystals. JETP Lett.

[3] Duan Q, Ma Y, Che M (2019). Fluorescent carbon dots as carriers for intracellular doxorubicin delivery and track. J Drug Deliv Sci Technol.

[4] Zrazhevskiy P, Gao X (2009). Multifunctional quantum dots for personalized medicine. Nano Today.

[5] Sahu A, Kumar D (2022). Core-shell quantum dots: a review on classification, materials, application, and theoretical modeling. J Alloys Compd.

[6] Phafat B, Bhattacharya S (2023). Quantum dots as theranostic agents: recent advancements, surface modifications, and future applications. Mini Rev Med Chem.

[7] Sathe KP, Garud NS, Bangar VB, Gadakh NR (2022). A review on quantum dots (QDs). J Adv Sci Res.

[8] Ye F, Barrefelt A, Asem H (2014). Biodegradable polymeric vesicles containing magnetic nanoparticles, quantum dots and anticancer drugs for drug delivery and imaging. Biomaterials.

[9] Yao X, Niu X, Ma K, Huang P, Grothe J, Kaskel S, Zhu Y (2017). Graphene quantum dots-capped magnetic mesoporous silica nanoparticles as a multifunctional platform for controlled drug delivery, magnetic hyperthermia, and photothermal therapy. Small.

[10] Cai X, Luo Y, Zhang W, Du D, Lin Y (2016). pH-sensitive ZnO quantum dots-doxorubicin nanoparticles for lung cancer targeted drug delivery. ACS Appl Mater Interfaces.

[11] Bharali DJ, Mousa SA (2010). Emerging nanomedicines for early cancer detection and improved treatment: current perspective and future promise. Pharmacol Ther.

[12] Delehanty JB, Boeneman K, Bradburne CE, Robertson K, Medintz IL (2009). Quantum dots: a powerful tool for understanding the intricacies of nanoparticle-mediated drug delivery. Expert Opin Drug Deliv.

[13] Iannazzo D, Pistone A, Salamò M (2017). Graphene quantum dots for cancer targeted drug delivery. Int J Pharm.

[14] Nigam P, Waghmode S, Louis M, Wangnoo S, Chavan P, Sarkar D (2014). Graphene quantum dots conjugated albumin nanoparticles for targeted drug delivery and imaging of pancreatic cancer. J Mater Chem B.

[15] Li Z, Xu W, Wang Y (2015). Quantum dots loaded nanogels for low cytotoxicity, pH-sensitive fluorescence, cell imaging and drug delivery. Carbohydr Polym.

[16] Abbaspourrad A, Datta SS, Weitz DA (2013). Controlling release from pH-responsive microcapsules. Langmuir.

[17] Peer D, Karp JM, Hong S, Farokhzad OC, Margalit R, Langer R (2007). Nanocarriers as an emerging platform for cancer therapy. Nat Nanotechnol.

[18] Sui X, Luo C, Wang C, Zhang F, Zhang J, Guo S (2016). Graphene quantum dots enhance anticancer activity of cisplatin via increasing its cellular and nuclear uptake. Nanomedicine.

[19] Qian C, Yan P, Wan G, Liang S, Dong Y, Wang J (2018). Facile synthetic photoluminescent graphene quantum dots encapsulated β-cyclodextrin drug carrier system for the management of macular degeneration: detailed analytical and biological investigations. J Photochem Photobiol B.

[20] Ke Y (2012). Recent patents on quantum dot engineering for biomedical application. Recent Pat Biomed Eng.

